# Enhanced Thermal Conductivity of Epoxy Composites Filled with 2D Transition Metal Carbides (MXenes) with Ultralow Loading

**DOI:** 10.1038/s41598-019-45664-4

**Published:** 2019-06-24

**Authors:** Ruiyang Kang, Zhenyu Zhang, Liangchao Guo, Junfeng Cui, Yapeng Chen, Xiao Hou, Bo Wang, Cheng-Te Lin, Nan Jiang, Jinhong Yu

**Affiliations:** 1 Key Laboratory for Precision and Non-Traditional Machining Technology of Ministry of Education, Dalian University of Technology, Dalian, 116024 China; 2 Key Laboratory of Marine Materials and Related Technologies, Zhejiang Key Laboratory of Marine Materials and Protective Technologies, Ningbo Institute of Materials Technology and Engineering, Chinese Academy of Sciences, Ningbo, 315201 China

**Keywords:** Nanocomposites, Nanocomposites, Two-dimensional materials, Two-dimensional materials

## Abstract

With the development of electronic devices such as integrated circuits toward the continual increase in power density and consumption, the efficient heat dissipation and low thermal expansion of materials become one of the most important issue. However, conventional polymers have the problem of poor thermal dissipation performance, which hinder application for electronic devices. In this work, the two-dimensional material, MXene (Ti_3_C_2_), is used as the reinforcement additive to optimize the thermal properties of polymers. We reported the preparation of multilayer Ti_3_C_2_ MXene by HF etching method and obtained few-layer Ti_3_C_2_ MXene by simple ultrasonication. Meanwhile, Ti_3_C_2_/epoxy composites were prepared by a solution blending method. The results show that the thermal properties of the composites are improved in comparison with the neat epoxy. Thermal conductivity value (0.587 W/mK) of epoxy composite with only 1.0 wt% Ti_3_C_2_ MXene fillers, is increased by 141.3% compared with that of neat epoxy. In addition, the composite presents an increased glass transition temperature, high thermal stability and lower coefficient of thermal expansion. This work is of great significance for the research of high-performance composite materials.

## Introduction

As electronic devices towards more integrated and lightweight, efficient heat dissipation is critical to enhance the longevity of the device^1–3^. Meanwhile, in the field of electronic packaging, it’s necessary for polymer composites to improve the thermal conductivity meanwhile lower thermal expansion coefficient^4^. In order to improve the thermal conductivity of polymers, a method of adding high content of fillers into the polymer matrix is employed in most previous works. The method often results in poor mechanical properties, high density and high costs^5^. These adverse consequences make it difficult for polymer composites to meet the requirements of industry application. Therefore, it is very meaningful to research on the preparation of polymer composites with high thermal conductivity and ultralow fillers loading.

Nowadays, two-dimensional materials have attracted more and more attention since the discovery of graphene^6^. Two-dimensional materials possess the greatly high aspect ratios and the thicknesses corresponding to a few atomic layers^7–10^. As a novel type of two-dimensional transition metal carbides or carbonitrides, MXenes possess a graphene-like two-dimensional structure and synthesized by HF selectively etching away A atom layers of the MAX phases^11,12^. And they possess a unique two-dimensional layered structure, high specific surface area, glorious thermal properties, outstanding adsorption properties, excellent mechanical properties and electrical properties. Thus, MXenes have been used in many fields such as energy storage, catalysis, adsorption, hydrogen storage, sensors and so on^13–27^. Up to now, many reported literatures explore the use of MXenes in energy storage applications, conductivity enhancers of polymers and reinforcement additives for the mechanical properties of polymers^27–32^. While, so far, few studies have considered MXenes as a material ever to optimize the thermal properties of polymers^33^.

Epoxy resin is high-performance engineering thermosetting plastic and possesses various outstanding properties, such as good chemical inertness, high wear resistance, excellent adhesive property, outstanding mechanical properties and so on. Hence, it is widely utilized in the fields of electronic products, high-voltage electrical equipment, petrochemicals and anti-corrosion pipeline valves^33–35^. However, the intrinsically low thermal conductivity and relatively inferior coefficient of thermal expansion limit its application^36,37^. Therefore, it is necessary to improve the thermal properties for epoxy resin. As one of the useful strategies for improving the thermal properties of polymers, adding reinforcement additives into polymers is often used. Among reinforcement additives, nanomaterials are advantageous for improving some properties of polymers due to large specific surface area and other unique properties^26^. By far, a lot of nanomaterials have been researched as fillers and reported in previous works, such as graphene^38,39^, boron nitride^40–43^, and silicon carbide nanowires^44,45^. These works demonstrate that the thermal, mechanical and electrical property can be significantly enhanced by addition of nanomaterials. In recent years, numerous two-dimensional materials have aroused a great deal of interest and triggered many works on improving thermal properties of polymers^6,46–50^. However, there is no study yet that using Ti_3_C_2_ MXene as additives to improve the thermal properties of epoxy resin. Hence, the studies on epoxy composites with Ti_3_C_2_ MXene are of theoretical and practical significance.

In this work, multilayer Ti_3_C_2_ MXene was prepared by directly etching MAX phase (Ti_3_AlC_2_) with HF. Afterwards, few-layer Ti_3_C_2_ MXene was obtained from multilayer Ti_3_C_2_ MXene by simple ultrasonication and was added into epoxy matrix to fabricate Ti_3_C_2_/epoxy composites by a solution blending method. As a result, the fabricated composites with ultralow loading showed higher thermal conductivity, glass transition temperature (T_g_), thermal stability and lower coefficient of thermal expansion (CTE) compared with that of neat epoxy.

## Materials and Methods

### Materials

Hydrogen fluoride (HF, 40%) and anhydrous ethanol were purchased from Sinopharm Chemical Reagent Co., Ltd. (Shanghai, China). Ti_3_AlC_2_ powder was obtained from Forsman Scientific Co., Ltd. (Beijing, China). Cycloaliphatic epoxy resin (6105, DOW Chemicals) used in the study was purchased from Shanghai Liyi Science & Technology Development, China. The curing agent, Methylhexahydrophthalic anhydride, was brought from Zhejiang Alpharm Chemical Technology Co., Ltd. (Zhejiang, China). Neodymium(III) 2,4-pentanedionate (Nd(III)acac) was provided by Aldrich Chemicals.

### Preparation of Ti_3_C_2_ MXene

Ti_3_C_2_ MXene was synthesized by exfoliating the MAX phases with hydrogen fluoride solution. The specific steps are showing as follows: first of all, 10.0 g Ti_3_AlC_2_ powder was slowly added into 100 ml 40% hydrogen fluoride solution in a polytetrafluoroethylene container. At room temperature, the mixture was kept being stirred for 3 h. Afterward, 300 ml deionized water was slowly added into the resulting mixture to get the diluted suspension. Subsequently, the suspension was centrifuged at 4000 rpm for 15 min and the supernatant was discarded to collect the sediment. The sediment was washed with deionized water until the pH was close to 7^51,52^. And then, the washed powder was dried under vacuum conditions at 100 °C for 6 h. To obtain few-layer Ti_3_C_2_ MXene, 2.5 g dried powder was added to 400 ml distilled water in a 500 ml glass beaker and was ultrasonicated for 2 h. The obtained suspension was centrifuged at 2000 rpm for 15 min. The supernatant was separated from the sediment powders and a black colloidal suspension was obtained. Finally, the colloidal suspension was filtered onto a 0.22 mm pore-size polypropylene membrane and was dried under vacuum conditions at 100 °C for 24 h to obtain few-layer Ti_3_C_2_ MXene powder.

### Preparation of epoxy composites

**Figure 1:**
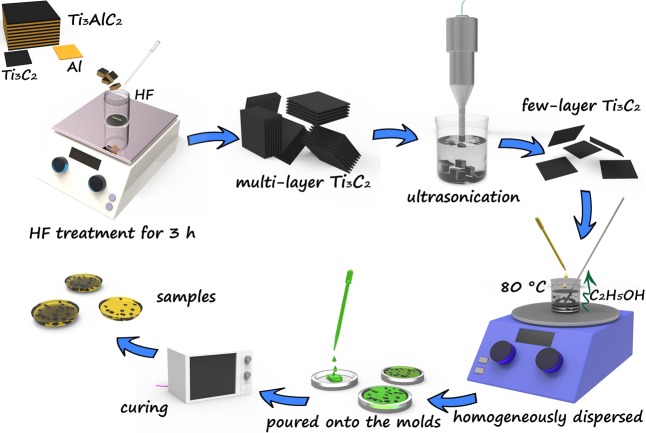
Schematic illustration of the preparation process of Ti_3_C_2_/epoxy composites.

The epoxy composites with varying contents of Ti_3_C_2_ MXene fillers were prepared as following steps. Firstly, Nd(III)acac was added to the epoxy resin, with the weight ratio to be 1:1000 (Nd(III)acac: epoxy resin). And the mixture was kept being stirred at 80 °C for 3 h. The resulting homogeneous solution was then cooled to room temperature. Secondly, 0.2, 0.4, 0.6, 0.8 and 1.0 wt% of Ti_3_C_2_ MXene (the weight ratio means the weight of Ti_3_C_2_ MXene to the weight of the epoxy composite) was added respectively into a certain amount of ethanol (1 mg of Ti_3_C_2_ MXene per 1 ml ethanol) and was then ultrasonicated for 1 h to form a homogeneously dispersed suspension. Subsequently, the pre-prepared epoxy resin was added into the suspension. Meanwhile, the mass of the mixture was measured and recorded. The mixture was then kept being stirred with an electric stirrer at 80 °C for 4 h. After that, the mixture was weighed every ten minutes and the mass difference was calculated. When the mass difference is equal to the mass of the added ethanol (the mass error ≤0.4%) and remains unchanged, the ethanol is considered to be evaporated completely. The real photos of the obtain mixture are shown in Fig. S1. Thirdly, curing agent was added into the mixture, with the weight ratio to be 95:100 (curing agent: the mixture) and was stirred for 15 min. Afterwards, the mixture was degassed under vacuum conditions at 50 °C for 2 h. Finally, the mixture was poured onto a preheated molds treated with a release agent and was pre-cured in an oven at 135 °C for 2 h. Subsequently, the temperature was adjusted to 165 °C and held for 14 h to achieve the sample post-curing process. After cooling to the ambient temperature, the epoxy composites were polished to obtain desired shapes. For comparison, the neat epoxy resin was prepared according to the above preparation process. As a convenience, Ti_3_C_2_/epoxy composites were used to represent the epoxy composites with Ti_3_C_2_ MXene fillers. The real photos of Ti_3_C_2_/epoxy composites and neat epoxy are exhibited in Fig. S2. And the schematic diagram of the preparation procedure is shown in Fig. 1.

### Characterization

A field emission scanning electron microscope (FE-SEM, Quanta 250, USA) equipped with energy dispersive spectrometer was used to characterize the surface morphology of the Ti_3_C_2_ MXene and the fractured surfaces of epoxy samples, with an accelerating voltage of 10 kV. To observe the nature of Ti_3_C_2_ MXene bonding to epoxy, samples were quenched in liquid nitrogen and then broken to obtain the fracture surfaces. Besides, to avoid the charge accumulation, a thin layer of gold was sprayed on the fracture surfaces. JEOL JEM2100 transmission electron microscopy (TEM) with an accelerating voltage of 200 kV was used to observe the microstructures of ultrathin Ti_3_C_2_ MXene nanosheets. The Ti_3_C_2_ MXene suspension was dropt onto carbon-coated copper grids and dried in a vacuum oven to fabricate the samples for TEM. A scanning probe microscope (Dimension 3100, Vecco, USA) was used to determine the thickness of exfoliated Ti_3_C_2_ MXene. A powder diffractometer (D8 ADVANCE, Bruker, Germany) with CuKα radiation was used to acquire the X-ray diffraction (XRD) patterns of Ti_3_C_2_ MXene. The Raman spectra was performed on a Raman spectrometer (Renishaw inVia Reflex, Renishaw plc, UK). Fourier transform infrared (FT-IR) spectra were obtained using a FTIR (Nicolet 6700, Thermal scientific Inc, USA) between 400 and 4000 cm^−1^. X-ray photoelectron spectra (XPS) was conducted by means of an X-ray photoelectron spectrometer (Axis Ultra DLD, Kratos Analytical Ltd., UK). A flash thermal conductivity meter (LFA 467, NETZSCH, Germany) was used for measurements of the thermal diffusivity (α, mm^2^/s) and the size of each measured sample is 10 (length) × 10 (width) × 0.8 mm (thickness). The thermal conductivity (λ, W/mK) of Ti_3_C_2_/epoxy composites and neat epoxy was calculated through the Eq. (λ = α × ρ × Cp, α is the thermal diffusivity, ρ is the density and Cp is the specific heat capacity). The density (ρ, g/cm^3^) was measured through liquid displacement method. The specific heat capacity (Cp, J/gK) and the glass transition temperature (T_g_) were obtained by differential scanning calorimetry (DSC). And the testing equipment is a differential scanning calorimeter (Pyris Diamond DSC, Perkin-Elmer, USA). Thermal gravimetric analysis (TGA) was conducted by using a TGA 209 F3 (NETZSCH, Germany). Composites and Ti_3_C_2_ MXene were measured in the temperature range of 50–800 and 50–1000 °C, respectively. Besides, all the measurements were conducted under a nitrogen atmosphere and the heating rate is 10 °C/min. The CTE measurements were conducted on a thermal mechanical analyzer (TMA 402 F1/F3, NETZSCH, Germany) from 40 to 180 °C at a heating rate of 5 °C/min. An infrared camera (Ti400, Fluke, U.S.A.) was used to take IR-photos.

## Results and Discussion

### Characterization of Ti_3_C_2_ MXene

**Figure 2:**
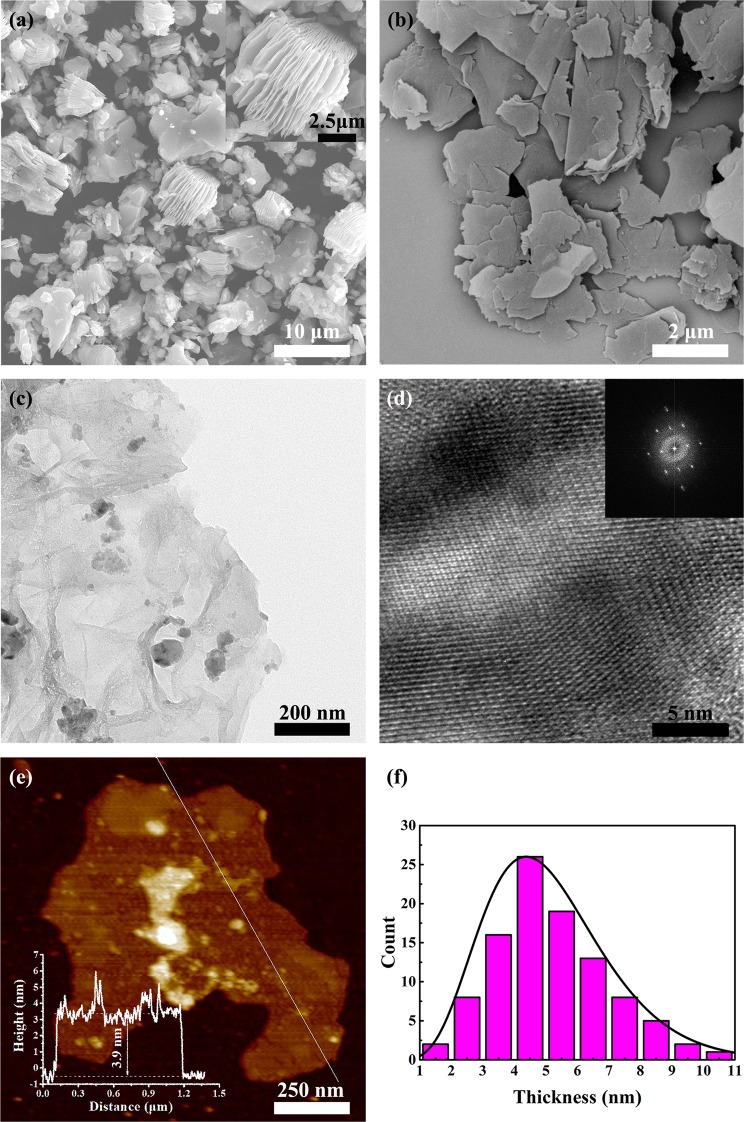
FE-SEM images of (**a**) multilayer Ti_3_C_2_ MXene and (**b**) few-layer Ti_3_C_2_ MXene, the high-resolution FE-SEM image of multilayer Ti_3_C_2_ MXene is shown in the inset of (**a**). (**c**) TEM and (**d**) higher-resolution TEM images of few-layer Ti_3_C_2_ MXene, the inset of (**d**) is the corresponding fast Fourier transform (FFT) of the thin Ti_3_C_2_ MXene nanosheet. (**e**) The AFM image of Ti_3_C_2_ MXene nanosheets. The inserted image exhibits the thickness of single Ti_3_C_2_ MXene nanosheet. (**f**) Histograms of measured value for Ti_3_C_2_ MXene nanosheets thickness.

As shown in Fig. 2(a,b), the morphology of the Ti_3_C_2_ MXene was observed by FE-SEM. Figure 2(a) shows the SEM image of multilayer accordion-like Ti_3_C_2_ MXene^53^. It is found that Ti_3_C_2_ MXene obtained by HF directly etching MAX phase has stacked layers and the thickness of Ti_3_C_2_ MXene is approximately 10 μm. From the inserted higher magnification image, the gaps between layers are of different sizes and the thickness of 1 μm corresponds to approximately 11 layers. Figure 2(b) presents that few-layer Ti_3_C_2_ MXene is mainly composed of ultrathin Ti_3_C_2_ MXene nanosheets and the lateral size of the Ti_3_C_2_ MXene nanosheets is approximately 2 μm. The results indicate that ultrasonication is effective for converting multi-layered Ti_3_C_2_ MXene into few-layer Ti_3_C_2_ MXene. Besides, the energy dispersive spectroscopy (EDS) of Ti_3_C_2_ Mxene was shown in Fig. S3. Figure 2(c,d) are the TEM images of few-layer Ti_3_C_2_ MXene. And the Fig. 2(c) demonstrates that the exfoliated Ti_3_C_2_ MXene nanosheets are quite thin and transparent. From a high-resolution TEM image, Fig. 2(d), the lattice of Ti_3_C_2_ can be observed clearly, which illustrates that Ti_3_C_2_ MXene is a kind of crystal. The inset of Fig. 2(d) is the corresponding fast Fourier transform (FFT) of the thin Ti_3_C_2_ MXene nanosheet and it obviously signifies that Ti_3_C_2_ MXene has hexagonal structure, which is the same with Ti_3_AlC_2_ (MAX phase)^11,54^. An AFM image of Ti_3_C_2_ MXene nanosheets is shown in the Fig. 2(e) and the inserted image shows that the thickness of a Ti_3_C_2_ MXene nanosheet is about 3.9 nm. In addition, the AFM image also proves that Ti_3_C_2_ MXene nanosheets are quite thin and flat, which is consistent with previous analysis of SEM and TEM. As shown in Fig. 2(f), we measured the thickness of 100 nanosheets and conducted a simple statistical analysis. The histogram reflects that the average thickness of Ti_3_C_2_ MXene nanosheets is 5.1 nm.

**Figure 3:**
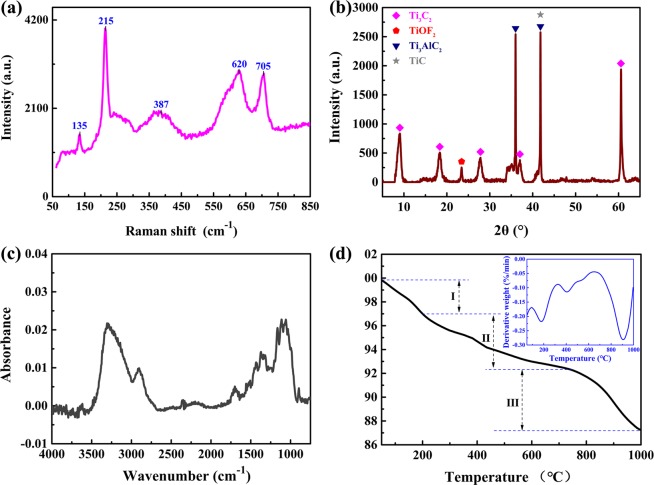
(**a**) Raman spectrum, (**b**) XRD pattern, (**c**) FTIR spectrum, and (**d**) Thermal gravimetric analysis (TGA) curve of Ti_3_C_2_ MXene. The differential thermal gravimetric (DTG) curve of Ti_3_C_2_ MXene is presented in the inset of (**d**).

Raman spectrum of the Ti_3_C_2_ MXene is shown in Fig. 3(a). The Raman peaks at 215, 387, 620 and 705 cm^−1^ are the characteristic peaks of Ti_3_C_2_ MXene^11,55^. The peaks at 215 and 705 cm^−1^ are assigned to the Ti-C and C-C vibrations (A1_g_ symmetry) of the oxygen-terminated Ti_3_C_2_O_2_. The peak at 387 cm^−1^ is attributed to the O atoms E_g_ vibrations. The peak at 620 cm^−1^ comes mostly from Eg vibrations of the C atoms in the OH-terminated Ti_3_C_2_. The above results illustrate that there are some surface terminations on the surface of Ti_3_C_2,_ such as -OH and -O. Additionally, the incompletely etched Ti_3_AlC_2_ brings about a sharp peak at around 135 cm^−1^ ^56^.

The XRD pattern of theTi_3_C_2_ MXene is shown in Fig. 3(b). Previous literature reports that the characteristic (002) peak and (004) peak of Ti_3_AlC_2_ are at 9.7° and 19.4°, respectively. However, it is obvious that the (002) peak and (004) peak shift to low angle, 9.1° and 18.3°, which is attributed to the newly synthesized Ti_3_C_2_ MXene^11,51^. In addition, the peaks appearing at 37° and 61.5° belong to Ti_3_C_2_ MXene. The shift and broadening of (002) peak mean that the d-spacing of Ti_3_C_2_ MXene is increased and the thickness of Ti_3_C_2_ MXene layers is declined^57,58^. The XRD peak at 23.4° is corresponding to the (100) peak of TiOF_2,_ which is formed from a reaction between Ti_3_C_2_ MXene with -F and -OH/-O terminating groups^59^. The (111) peak at 36° and (200) peak at 41.7° are assigned to TiC impurity^60^. Besides, the peak at 41.7° partly testifies the presence of residual Ti_3_AlC_2._ It is notable that the characteristic (104) peak (39.2°) of Ti_3_AlC_2_ disappears, which indicates that the HF etch of Ti_3_AlC_2_ (MAX phase) is efficient^52^.

In order to characterize the functional groups on the surface of Ti_3_C_2_ MXene, the FTIR spectrum of theTi_3_C_2_ MXene is shown in Fig. 3(c). The peaks at 1700 and 3300 cm^−1^ demonstrate the existence of crystal water. The peak at 1645 cm^−1^ reveals the existence of liquid water^13^. There are four peaks at 1110, 1055, 1020 and 990 cm^−1^, due to the stretching vibrations of C-OH^61,62^. The peaks at 1147 and 1160 cm^−1^ are respectively assigned to the symmetric stretching of CH_3_ and CH_2_. The peaks appear at 1210 and 1278 cm^−1^, corresponding to the antisymmetric stretching of CH_2_ and CH_3_. Additionally, there are some characteristic peaks at 1315, 1340, 1370 and 2900 cm^−1^, which are associated with the C-H band of CH_x_^63^. Particularly, the characteristic peak at 2900 cm^−1^ is assigned to the stretching vibration of C-H. The FTIR results agree well with the foregoing Raman spectrum and XRD.

For the research of the thermal stability of Ti_3_C_2_ MXene, the TGA and DTG curve of Ti_3_C_2_ MXene are exhibited in Fig. 3(d). The TGA curve is divided into three stages. In the first stage (50–200 °C), the weight loss is about 2.8%, which is caused by the loss of physically adsorbed water and HF on Ti_3_C_2_ MXene surface^64–67^. Due to the loss of OH groups attached on Ti_3_C_2_ MXene surface, the weight loss is approximately 4.9% in the second stage (200–780 °C). In the third stage (780–1000 °C), the weight loss is about 5.2%, which is caused by the loss of chemically bonded F and O groups^68^.

**Figure 4:**
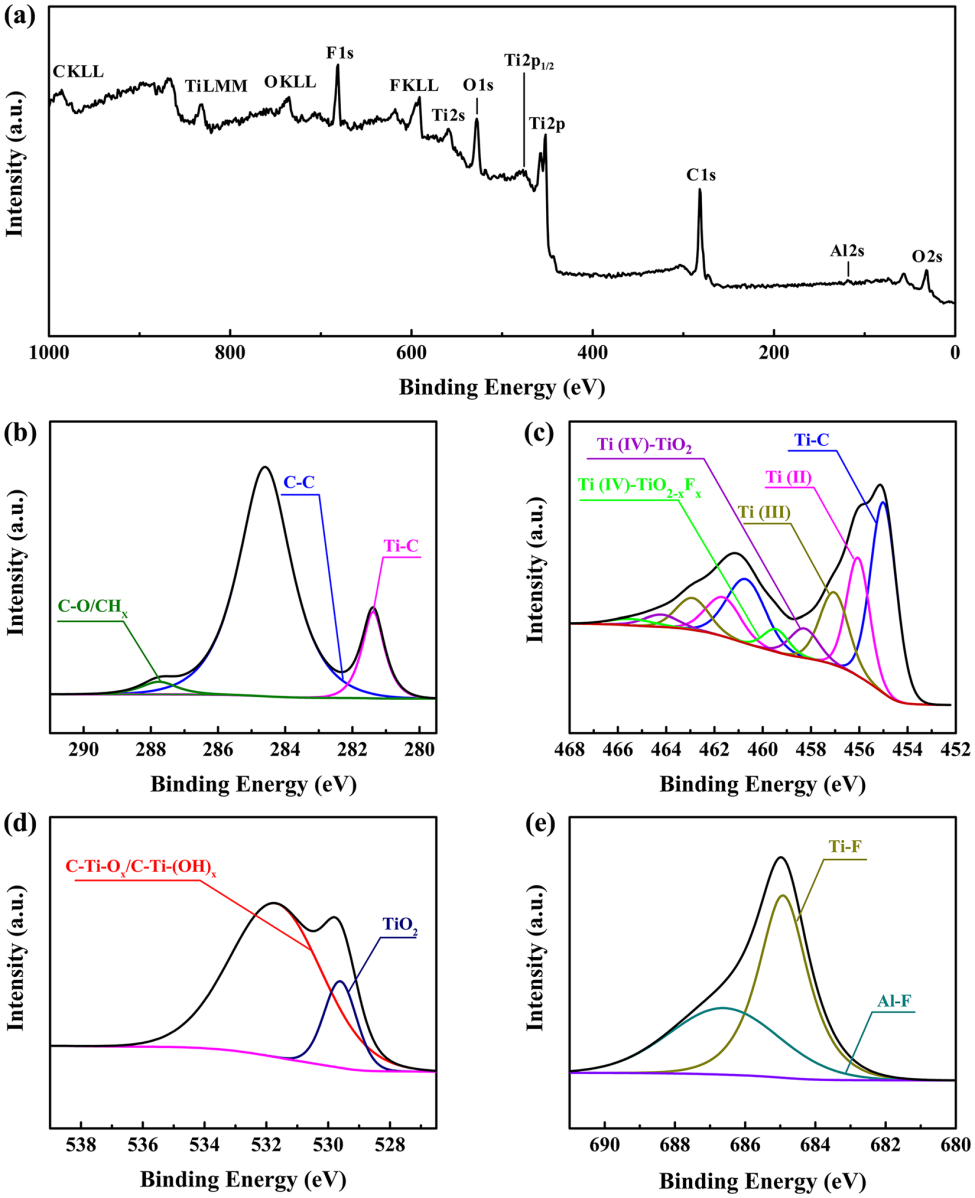
XPS spectra: (**a**) survey scan spectrum, (**b**) region C 1s, (**c**) region Ti 2p, (**d**) region O 1s and (**e**) region F 1s of Ti_3_C_2_ MXene.

To study the surface chemical compositions of Ti_3_C_2_ MXene in detail, XPS analysis was conducted and shown in Fig. 4, which is recorded for O 2s, Al 2s, C 1s, Ti 2p, Ti 2p1/2, O 1s, Ti 2s, F KLL, F 1s, O KLL, Ti LMM and C KLL^69^. In particular, from Fig. 4(a), the survey scan spectrum of Ti_3_C_2_ MXene, it is known that the peaks at around 284, 455, 532 and 685 eV respectively correspond to C 1s, O 1s, Ti 2p and F 1s. The region C 1s of Ti_3_C_2_ MXene is shown in Fig. 4(b) and fit by three peaks. The peak at 281.4 eV is deconvoluted into the component assigned to C-Ti-T_x_, such as Ti_3_C_2_O_x_, Ti_3_C_2_(OH)_x_, Ti_3_C_2_F_x_ and Ti_3_C_2_OH-H_2_O^70,71^. The peak at 284.7 eV is assigned to graphitic C-C and the peak at 287.6 eV is assigned to CH_x_ or C-O^72^. Figure 4(c) is the region Ti 2p of Ti_3_C_2_ MXene. The peaks at binding energy values of 455, 455.8, 457.2, 458.6 and 459.3 eV are assigned to Ti-C, Ti(II), Ti(III), TiO_2_ and TiO_2-x_F_x_, respectively^70,71,73,74^. The presence of Ti(II) and Ti(III) is due to the formation of Ti_3_C_2_O_X_, Ti_3_C_2_(OH)_x_ and Ti_3_C_2_OH-H_2_O. Figure 4(d) is the region O 1s of Ti_3_C_2_ MXene sample, which is divided into two peaks. The peak at 529.9 eV corresponds to TiO_2_ and the peak at 532 eV is assigned to C-Ti-O_x_ or C-Ti-(OH)_x_^69,75^. The region F 1s of Ti_3_C_2_ MXene sample is shown in Fig. 4(e), which is fit by two peaks corresponding to two components. The major component in the region F1s is Ti-F, whose fitted peak is at 685 eV^76^. The other occurring at 686.4 eV is assigned to Al-F^74^. The above results indicate that the terminations of the Ti_3_C_2_ MXene surface are various and also demonstrate the existence of some surface terminations, such as -O, -OH and -F.

### Microstructure of composites

**Figure 5:**
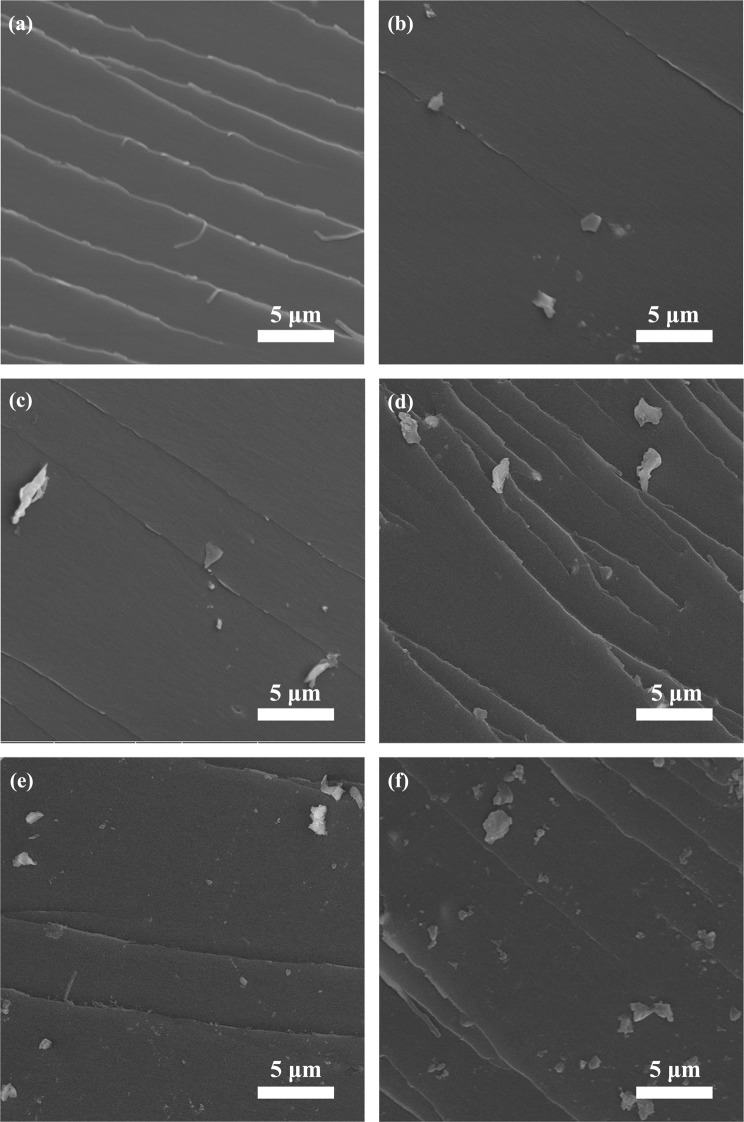
FE-SEM images of the fractured surfaces of the Ti_3_C_2_/epoxy composites with different amounts of Ti_3_C_2_ MXene: (**a**) 0 wt%, (**b**) 0.2 wt%, (**c**) 0.4 wt%, (**d**) 0.6 wt%, (**e**) 0.8 wt% and (**f**) 1.0 wt%, respectively.

To further study the dispersion of Ti_3_C_2_ MXene in the epoxy matrix and its interfacial adhesion with epoxy matrix, a FE-SEM was used to investigate the microcosmic morphologies of the samples. The SEM images were shown in Fig. 5(a–f), which show microstructure of neat epoxy and its composites with 0.2, 0.4, 0.6, 0.8 and 1.0 wt% Ti_3_C_2_ MXene fillers, respectively. Figure 5(a) reveals that the stripes appearing on the fracture surface are like river shapes and the regions between stripes are very smooth. These stripes are typical characteristics of brittle thermosetting polymer. As is shown in Fig. 5(b–f), there are also some stripes on the fracture surfaces, but Ti_3_C_2_/epoxy composites possess rougher fracture surfaces compared with neat epoxy. Furthermore, it can be obviously observed that few-layer Ti_3_C_2_ MXene fillers are homogeneously distributed and inserted into the matrix, which reveals the strong interaction between few-layer Ti_3_C_2_ MXene fillers and the matrix. Meanwhile, as the content of the filler increases, more and more few-layer Ti_3_C_2_ MXene fillers appear on the fracture surfaces.

### Thermal properties of composites

**Figure 6:**
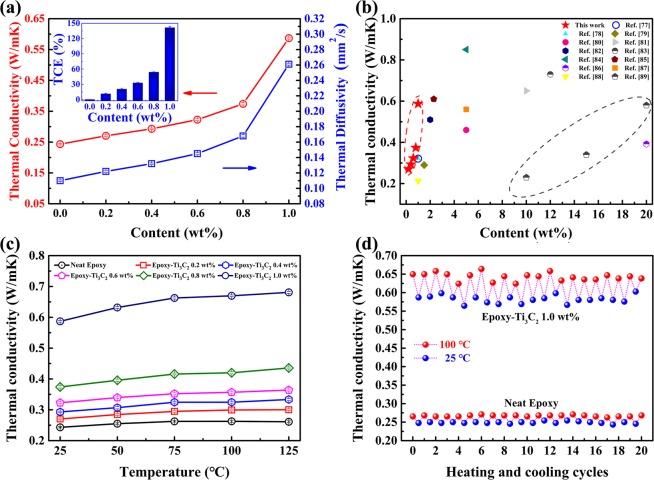
(**a**) Thermal diffusivity and thermal conductivity of neat epoxy and Ti_3_C_2_/epoxy composites. The thermal conductivity enhancement (TCE) was calculated and shown in the inset of (**a**). (**b**) Comparison of thermal conductivity results in our work with those of previous reports on epoxy composites. (**c**) Thermal conductivity of neat epoxy and Ti_3_C_2_/epoxy composites at different temperatures. (**d**) Heating and cooling cycles of neat epoxy and Ti_3_C_2_/epoxy composites.

Figure 6(a) reveals the heat conduction performance of Ti_3_C_2_/epoxy composites and neat epoxy. It can be clearly known that the heat conduction performance of these composites is gradually improved with an increase in the amount of Ti_3_C_2_ MXene fillers. The thermal diffusivity value and thermal conductivity value of neat epoxy are 0.110 mm^2^/s and 0.243 W/mK, respectively. The thermal diffusivity values of Ti_3_C_2_/epoxy composites with 0.2, 0.4, 0.6, 0.8 and 1.0 wt% Ti_3_C_2_ MXene fillers are 0.122, 0.132, 0.145, 0.168 and 0.261 mm^2^/s, respectively. And the corresponding thermal conductivity values of Ti_3_C_2_/epoxy composites are 0.270, 0.293, 0.323, 0.374 and 0.587 W/mK. The thermal conductivity enhancement (TCE) was calculated and shown in the inset of Fig. 6(a). The inset clearly shows that the thermal conductivity value of epoxy composite with 1.0 wt% Ti_3_C_2_ MXene filler is improved by 141.3% compared with that of neat epoxy. To properly evaluate heat conduction performance of the samples in this work, some thermal conductivity values of epoxy composites, which are reported in previous works. As shown in Fig. 6(b), at low loading, the experimental results in this work are much higher than that of the summarized works^77–89^. In addition, this work possesses comparable or even higher the thermal conductivity values compared with previous works, where filler loading is higher. It should be noted that the thermal conductivity value of the epoxy composite with 20 wt% few-layer boron nitride nanosheets is 0.58 W/mK and that of the epoxy composite with 5.0 wt% modified graphene nanosheets is 0.56 W/mK^87,89^. The results of those two works are lower than that of this wok (0.587 W/mK). In order to reasonably explain the improvement of thermal conductivity for this work, the influencing factors are analyzed as follows. The neat epoxy and its composites are amorphous polymers. Phonons, a quantum mode of vibration, play a key role in heat conduction of amorphous polymers. In this mode of heat conduction, the scattering of phonons determines the thermal resistance at low temperatures and influences the heat conduction performance. As known to all, neat epoxy is poor in crystal structure. And the molecular chains of the epoxy resin are randomly entangled, which causes a severe phonon scattering phenomenon^90^. Therefore, neat epoxy possesses low thermal conductivity. Addition of high thermal conductivity fillers, scattering of phonons and interfacial thermal resistance between fillers and matrix significantly affect the heat conduction performance of polymer composites. Ti_3_C_2_ MXene fillers are added into the epoxy matrix as reinforcement phase. As a bridge between epoxy molecular chains, Ti_3_C_2_ MXene promotes the transfer of heat between molecular chains, which could efficiently improve the heat conduction performance of epoxy composites^5,77,91,92^. Furthermore, the addition of Ti_3_C_2_ MXene fillers could reduce the randomness of the molecular chains around fillers and weaken the scattering of phonons in a degree. In addition, Ti_3_C_2_ MXene fillers homogeneously disperse in the epoxy matrix and surface terminations of Ti_3_C_2_ MXene are beneficial to forming the good interface compatibility between fillers and epoxy matrix, which reduce the interfacial thermal resistance.

In order to study the effect of temperature on heat conduction performance of the samples, the tests of thermal conductivity were conducted at 25, 50, 75, 100 and 125 °C, respectively, as shown in Fig. 6(c). On the whole, the thermal conductivity values of these samples are elevated with an increase in the amount of fillers, which is well matched with Fig. 6(a). For a single sample, the thermal conductivity value increases with the increase of the temperature. The heat conduction mechanism of opaque amorphous polymers is mainly phonon heat conduction^93^. Hence, the phenomenon of increased thermal conductivity values is explained by using Eq. (1):

1 $${\lambda }_{ph}=1/3\times v\times c\times l$$

where ${\lambda }_{ph}$ *v*, *c* and *l* are respectively the thermal conductivity, the movement velocity, the specific heat capacity and the mean free path of phonons. Within the test temperature range, the movement velocity (*v*) is only related to the density and elastic mechanics properties of the material itself, and it can be considered as a constant. The specific heat capacity (*c*) increases with temperature. And as the temperature rises, the volume of the composites and segmental mobility of molecular chains increases, resulting in a reduction in the degree of interaction or entanglement between the molecular chains, which facilitates the increase of the mean free path (*l*) of the phonons. Furthermore, the molecular chains around the fillers become more ordered with a rise of test temperature, which also leads to a slight increase in the free path. Hence, the thermal conductivity values of the composites may increase with temperature.

Moreover, the heating and cooling cycles of neat epoxy and Ti_3_C_2_/epoxy composite with 1.0 wt% Ti_3_C_2_ MXene fillers are shown in Fig. 6(d). The temperature alternately changed between 25 and 100 °C during the process. Within the twenty cycles, the thermal conductivity values of the samples almost maintained the original values and slight fluctuations occurred. The result suggests that the samples possess the stability of heat conduction within this temperature range^94^.

**Figure 7:**
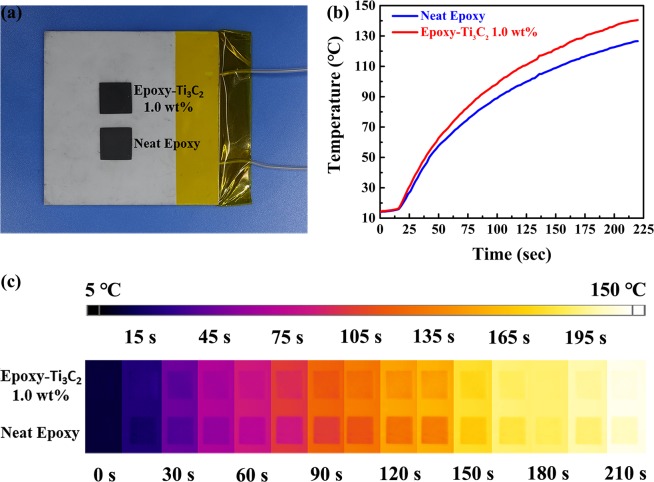
(**a**) The photo of a ceramic plate heater and the measured samples. (**b**) Heating curves and (**c**) infrared images of the measured samples.

For the purpose of visually comparing the heat conduction performance of epoxy composites with 1.0 wt% Ti_3_C_2_ MXene fillers and neat epoxy, the surface temperatures of the samples were recorded by an infrared camera and the results are presented in Fig. 7. Figure 7(a) is the picture of a ceramic plate heater and the measured samples. The size of each measured sample is 10 (length) × 10 (width) × 0.8 mm (thickness). Two samples were placed vertically with the composite on the top of the ceramic plate heater and the neat epoxy on the bottom. When the ceramic plate heater was connected with the power, the measured samples would be evenly heated and the surface temperatures of the samples would increase over time. To maintain the uniformity of heat conduction, the ceramic plate heater and the measured samples were sprayed with graphite before being heated. As shown in Fig. 7(b), by measuring the surface temperatures at the center of two samples as often as every two seconds, we obtained a series of temperature points and drew the heating curve. It is obvious that the heating curve of Ti_3_C_2_/epoxy composite is always above that of neat epoxy, demonstrating that it possess better heat conduction performance. Figure 7(c) shows the infrared images of the samples at different times. During the heating process, the surface temperature of the ceramic plate heater increased from 10 to 150 °C. The color of the neat epoxy is darker than that of Ti_3_C_2_/epoxy composite, indicating that the surface temperature of the neat epoxy is lower. Besides, it can also be seen that the color of the composite approximates that of the heater at 210 s.

**Figure 8:**
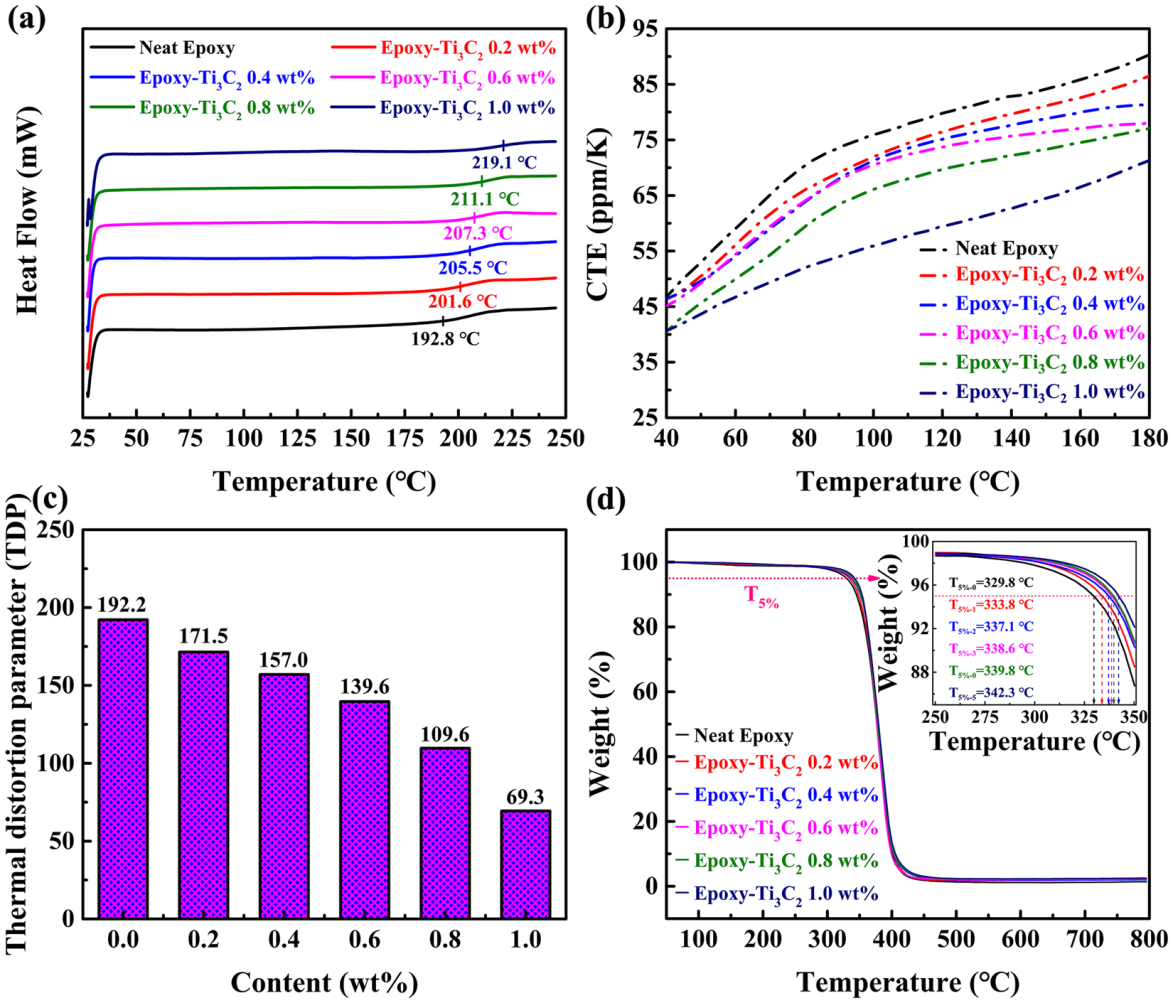
(**a**) DSC curves, (**b**) CTE curves, (**c**) TDP and (**d**) TGA curves of neat epoxy and Ti_3_C_2_/epoxy composites. Inset is the magnified curves of the TGA.

Figure 8(a) displays the glass transition temperatures (T_g_) of the samples. It can be observed that the T_g_s of these composites are gradually improved upon the addition of Ti_3_C_2_ MXene fillers. The T_g_ of neat epoxy is 192.8 °C. Meanwhile, those of the Ti_3_C_2_/epoxy composites with 0.2, 0.4, 0.6, 0.8 and 1.0 wt% Ti_3_C_2_ MXene fillers are 201.6, 205.5, 207.3, 211.1 and 219.1 °C, respectively. About the improvements of the T_g_s, some factors are considered as follows. The Ti_3_C_2_ MXene fillers added into the epoxy matrix can serve as physical interlock points and be intertwined by epoxy chains, which is conducive to restricting epoxy chains and reducing the motion of them^27^. Furthermore, the hydroxy groups of the Ti_3_C_2_ MXene fillers take part in the curing reaction so as to improve the crosslinking density^95^.

As shown in Fig. 8(b), the coefficients of thermal expansion (CTE) of neat epoxy and its composites were measured by a thermal mechanical analyzer from 40 to 180 °C. It can be seen that the coefficients of thermal expansion decrease with the increasing amount of the fillers within the investigated temperature range. Specifically, the CTE of neat epoxy is 46.7 ppm/K at 40 °C. And those of the epoxy composites with 0.2, 0.4, 0.6, 0.8 and 1.0 wt% Ti_3_C_2_ MXene fillers are 46.3, 46.0, 45.1, 41.0 and 40.7 ppm/K at 40 °C, respectively. In addition, the thermal strain curves are obtained and shown in Fig. S4. From the figure, it can be observed that the trend of the thermal strain curves is similar to that of CTE curves. When the temperature rises, the thermal movement of the molecular chains intensifies. The distance between the molecular chains also increase. These changes lead to an increase in the volume of epoxy. As mentioned in DSC section, adding the Ti_3_C_2_ MXene fillers into epoxy matrix can restrict epoxy chains and reduce the motion of them^96^. Therefore, the prepared epoxy composites exhibit lower CTE and thermal strain values compared with neat epoxy. In this work, TDP is introduced and used to indicate the comprehensive performance of engineering materials^4^. It is defined as the following Eq. (2):

2 $${\rm{TDP}}=\frac{CTE\,({K}^{-1})}{{T}_{c}\,(W{m}^{-1}\,{K}^{-1})}$$

The low TDP value of a sample means an excellent thermal property, indicating that the sample possesses relatively low CTE and high thermal conductivity. The TDP values of the Ti_3_C_2_/epoxy composites and neat epoxy are calculated and shown in Fig. 8(c). It can be clearly seen that the TDP decreases with an increasing amount of the fillers and that of the Ti_3_C_2_/epoxy composites with 1.0 wt% fillers is the lowest of all. The result illustrates the fact that Ti_3_C_2_ MXene is a kind of fine fillers to improve the thermal property of the epoxy matrix.

In order to further study the thermal stability of the samples, the thermal gravimetric analysis (TGA) was conducted and the TGA curves are shown in Fig. 8(d). Obviously, the degradation curves of the samples are similar, indicating that the degradation mechanism of the epoxy matrix has not been significantly changed due to the presence of Ti_3_C_2_ MXene. The decomposing temperatures occur at around 300 °C. However, perhaps it is because of small difference in filler content, the TGA curves are very close. It is difficult to accurately compare the decomposing temperatures. Hence, the temperatures at which composites degrade by 5% weight loss (T_5%_) were selected as characteristic thermal parameters to clearly compare the thermal stability of the samples. Meanwhile, the curves over the temperature range (250~350 °C) were amplified and shown in the inset. T_5%-0_, T_5%-1_, T_5%-2_, T_5%-3_, T_5%-4_ and T_5%-5_ respectively represent the characteristic thermal parameters of the epoxy composites with 0, 0.2, 0.4, 0.6, 0.8 and 1.0 wt% Ti_3_C_2_ MXene fillers. The values of the characteristic thermal parameters are 329.8, 333.8, 337.1, 338.6, 339.8 and 342.3 °C, respectively. The results show that the epoxy composites possess better thermal stability than neat epoxy resins. Because the molecular chains of the epoxy matrix are restricted by Ti_3_C_2_ MXene fillers and the thermal movement of them are weakened. In addition, during composite degradation, a Ti_3_C_2_ MXene char acts as a mass transport barrier and an isolator between the bulk polymer matrix and surface, where combustion occurs^97,98^. Therefore, the thermal stability of the epoxy composites is enhanced.

## Conclusions

In this work, few-layer Ti_3_C_2_ MXene was prepared by a simple and effective ultrasonic method to obtain. And adding the Ti_3_C_2_ MXene fillers into the epoxy matrix is proposed to prepare the epoxy composites possessing excellent thermal properties. The thermal conductivity of epoxy composite with 1.0 wt% Ti_3_C_2_ MXene is 0.587 W/mK and increased by 141.3% compared with that of neat epoxy. Meanwhile, the T_g_ and decomposing temperatures of epoxy composites are improved with the increasing Ti_3_C_2_ MXene fillers. Particularly, the T_g_ of the epoxy composite with 1.0 wt% Ti_3_C_2_ MXene is 219.1 °C, corresponds to a 13.7% improvement compared with that of neat epoxy. Moreover, the CTE and TDP values decrease with the increase of fillers content. Our work will pave the way for novel fundamental and application studies of MXene.

## Supplementary information

SUPPORTING INFORMATION-Enhanced Thermal Conductivity of Epoxy Composites Filled with 2D Transition Metal Carbides (MXenes) with Ultralow Loading
