# Supplementary material for: Enhanced Thermal Conductivity of Epoxy Composites Filled with 2D Transition Metal Carbides (MXenes) with Ultralow Loading
Source: Sci Rep. 2019 Jun 24;9:9135. doi: 10.1038/s41598-019-45664-4 (PMC6591414; doi:10.1038/s41598-019-45664-4)
Supplement: Supplementary file 1 — SUPPORTING INFORMATION-Enhanced Thermal Conductivity of Epoxy Composites Filled with 2D Transition Metal Carbides (MXenes) with Ultralow Loading [file 41598_2019_45664_MOESM1_ESM.docx]

**SUPPORTING INFORMATION**

**Enhanced Thermal Conductivity of Epoxy Composites Filled with 2D Transition Metal Carbides (MXenes) with Ultralow Loading**

Ruiyang Kang^a,b^, Zhenyu Zhang^a,*^, Liangchao Guo^a,b^, Junfeng Cui^a,b^, Yapeng Chen^b^, Xiao Hou^b^, Bo Wang^a,b^, Cheng-Te Lin^b^, Nan Jiang^b^, Jinhong Yu^b,*^

*^a^Key Laboratory for Precision and Non-Traditional Machining Technology of Ministry of Education, Dalian University of Technology, Dalian 116024, China E-mail address:* ***zzy@dlut.edu.cn***

*^b^Key Laboratory of Marine Materials and Related Technologies, Zhejiang Key Laboratory of Marine Materials and Protective Technologies, Ningbo Institute of Materials Technology and Engineering, Chinese Academy of Sciences, Ningbo 315201, China. E-mail address:* ***yujinhong@nimte.ac.cn***

**SUPPLEMENT**

**
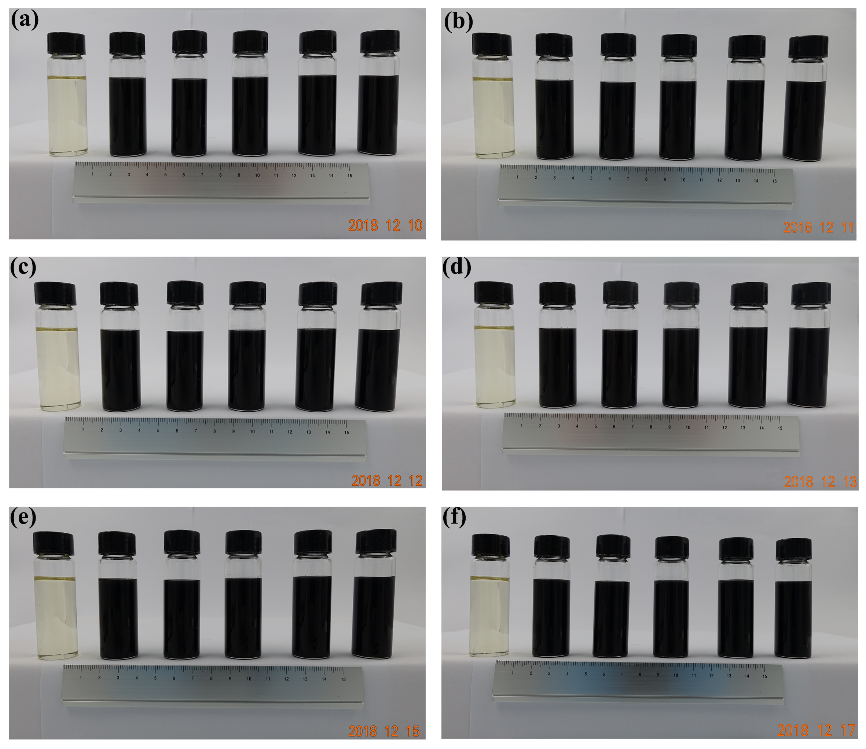
**

**Figure S1.** After statically placed for (**a**) 0, (**b**) 24, (**c**) 48, (**d**) 72, (**e**) 120 and (**f**) 168 h, the photos of the mixture before adding the curing agent.

In the preparation of neat epoxy and composites with different Ti_3_C_2_ MXene contents, 16 ml of each mixture before adding the curing agent was taken into a 20 ml glass bottle and was statically placed for a different period of time to judge whether the suspension was stable. The results are shown in **Fig S1**. In these photos, the mixtures (from left to right along the horizontal) is neat epoxy mixture and the mixture with 0.2, 0.4, 0.6, 0.8 and 1.0 wt% Ti_3_C_2_ MXene fillers, respectively. The neat epoxy mixture is a slightly yellow transparent liquid and the mixtures with Ti_3_C_2_ MXene fillers added are uniformly black liquids. **Figure S1(a-f)** respectively show the state of the mixtures after being statically placed for 0, 24, 48, 72, 120 and 168 h. It is obvious that there is no delamination and significant sedimentation phenomenon in the mixtures after being statically placed. However, the preparation time of the composite sample was not more than 48 h in the experiment. Therefore, the results indicate that the mixture is sufficiently stable for the preparation of uniform epoxy composites.


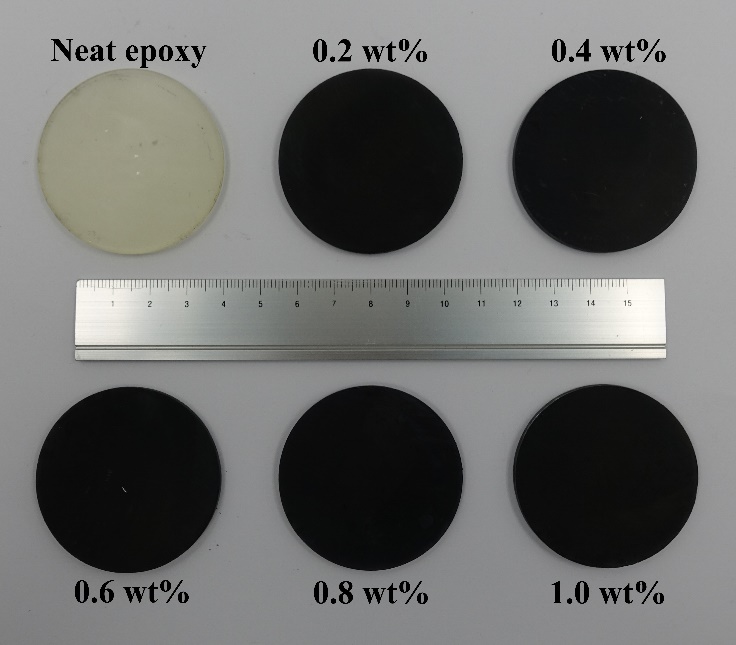


**Figure S2.** The photo image of neat epoxy and Ti_3_C_2_/epoxy composites.

The photo of Ti_3_C_2_/epoxy composites and neat epoxy without being sprayed with graphite is shown in **Fig S2**. Because the surface edges of the prepared Ti_3_C_2_/epoxy composites and neat epoxy are uneven, the prepared samples were polished slightly by using emery papers. As shown, the Ti_3_C_2_/epoxy composites are uniformly black and the neat epoxy is light yellow.


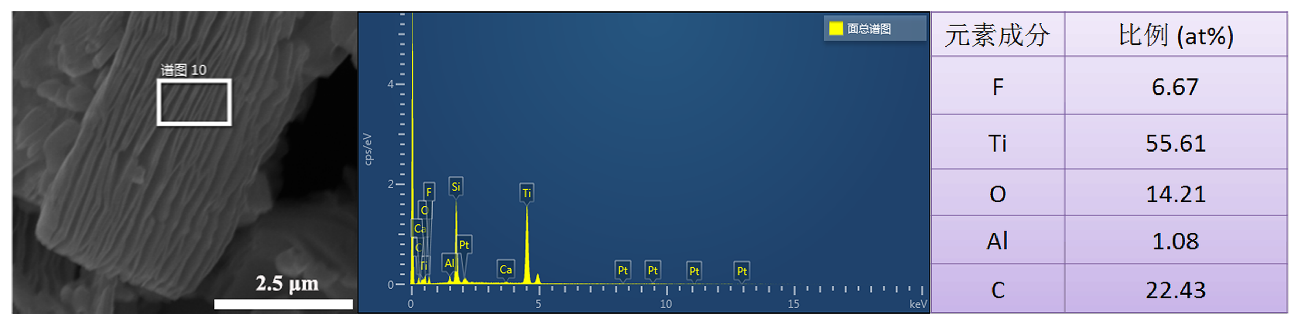


**Figure S3.** The SEM and energy dispersive spectroscopy (EDS) images of Ti_3_C_2_ Mxene.

The energy dispersive spectroscopy (EDS) of Ti_3_C_2_ Mxene was conducted and shown in **Fig. S3**. Because the observed sample is made on a silicon wafer, there is an [obvious](file:///D:\%E5%AD%A6%E4%B9%A0%E8%BD%AF%E4%BB%B6\%E6%9C%89%E9%81%93\Dict\7.5.0.0\resultui\dict\?keyword=obvious)signal of Si in the sample. Ignoring the signal of Si, there are also signals of F, Ti, O, Al and C elements in the sample. From the picture, it can be read that the content of Al is extremely low (1.08%) and the ratio of Ti to C is about at 3:2. The results clearly illustrate that the etching effect of hydrofluoric acid on the MAX phase is greatly significant.


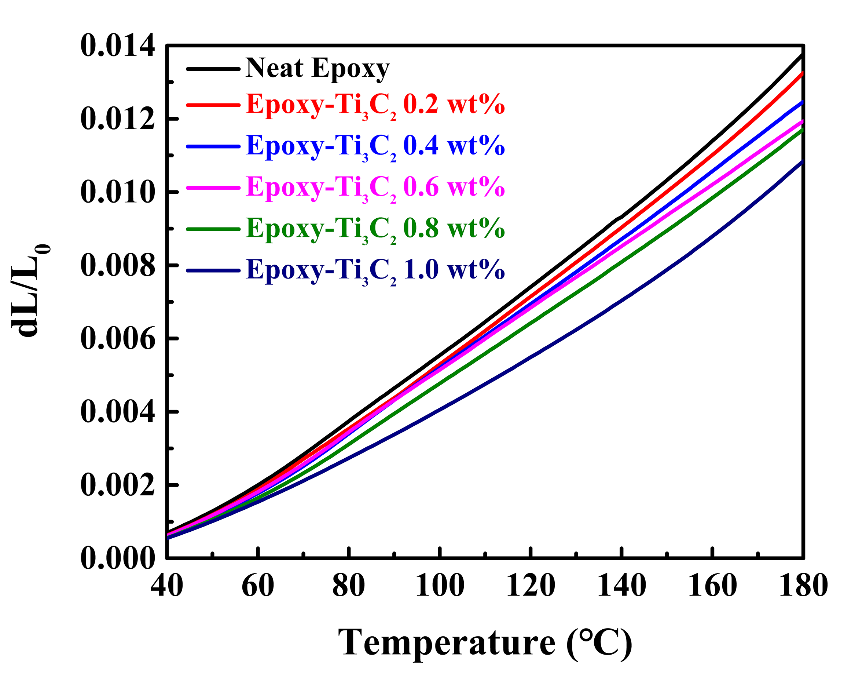


**Figure S4.** Thermal strain curves of neat epoxy and Ti_3_C_2_/epoxy composites.
